# PEPCONF, a diverse data set of peptide conformational energies

**DOI:** 10.1038/sdata.2018.310

**Published:** 2019-01-22

**Authors:** Viki Kumar Prasad, Alberto Otero-de-la-Roza, Gino A. DiLabio

**Affiliations:** 1 Department of Chemistry, University of British Columbia, Okanagan, 3247 University Way, Kelowna, British Columbia, V1V 1V7, Canada; 2 Department of Physical and Analytical Chemistry, Faculty of Chemistry, University of Oviedo, Oviedo, 33006, Spain; 3 Faculty of Management, University of British Columbia, Okanagan, 1137 Alumni Avenue, Kelowna, British Columbia, V1V 1V7, Canada

**Keywords:** Computational chemistry, Quantum chemistry

## Abstract

We present an extensive and diverse database of peptide conformational energies. Our database contains five different classes of model geometries: dipeptides, tripeptides, and disulfide-bridged, bioactive, and cyclic peptides. In total, the database consists of 3775 conformational energy data points and 4530 conformer geometries. All the reference energies have been calculated at the LC-ωPBE-XDM/aug-cc-pVTZ level of theory, which is shown to yield conformational energies with an accuracy in the order of tenths of a kcal/mol when compared to complete-basis-set coupled-cluster reference data. The peptide conformational data set (PEPCONF) is presented as a high-quality reference set for the development and benchmarking of molecular-mechanics and semi-empirical electronic structure methods, which are the most commonly used techniques in the modeling of medium to large proteins.

## Background & Summary

The structure and function of proteins are governed by the intermolecular interactions between their building blocks, amino acids. The accurate prediction of protein folding and ligand binding energetics depends on how well the computational modeling method employed captures the interactions between individual amino acids. For this reason, results obtained from the computational methods commonly employed to model proteins, such as force field and semi-empirical electronic structure methods, are usually compared to, and parametrized against, those obtained from higher-level computational methods. A database of peptide conformational energies is an ideal benchmark set for testing and parameterizing computational methods since conformational energies capture the interplay between bonded and non-bonded interactions that are present in proteins.

Similar sets to the one proposed in this work are available in the literature, but they tend to be small and focus on specific peptide interactions or otherwise focus exclusively on single amino acids. In 2008, Hobza and co-workers presented a benchmark database of conformational energies for a set of 76 conformers of four tripeptides and a dipeptide containing aromatic side chains^1^. The conformational energies were calculated at the CCSD(T)/complete-basis-set (CBS) level of theory and, in the same work, were used to assess lower-level quantum-mechanical (QM) methods. The reference data for a subset of Hobza’s set (named PCONF) was updated by Smith and co-workers^2^, and later by Goerigk and co-workers^3^. Wilke *et al.* proposed a set of conformational energies for cysteine known as CYCONF^4^, eight conformational energies of tetrapeptide conformers were proposed by Goerigk *et al*.^5^, and Ropo *et al.* presented a conformer data set of capped and uncapped versions of proteinogenic amino acids and their interactions with divalent cations evaluated at ‘PBE + vdW’ level of theory^6^. More recently, Martin and co-workers re-optimized the conformer structures of twenty proteinogenic amino acids from a previously published set by Yuan, Mills, Popelier, and Jensen (the YMPJ database)^7,8^. These structures were then used to generate a new conformational energy database of isolated amino acid monomers containing 466 data points. A database of macrocyclic conformers, called MPCONF196, has recently been published^9^. The MPCONF196 set contains conformational energies of eight macrocyclic compounds including cyclic peptides of varying sizes. To our knowledge, MPCONF196 is the only set in the literature that considers cyclic peptides. Several of the data sets described above have been compiled into supersets. Hobza’s 2008 data set was included as a subset of the MPCONF196 benchmark database^1,9^. Similarly, the CYCONF, PCONF, TPCONF, and YMPJ sets of conformational energies were incorporated in the GMTKN databases by Grimme and co-workers^3,10,11^.

To best of our knowledge, an extensive database of polypeptide conformations is not yet available in the literature. It is likely that the absence of a comprehensive data set rests on the fact that structural complexity and the computational cost of obtaining reference-quality data increases with system size. A comprehensive set of data that contains reference conformational energies on a diversity of small peptides would provide valuable information to those engaged in the development of atomistic computational methods for protein modeling. Producing such a database of conformational energies of diverse polypeptides would ensure a uniform high-quality standard in the reference data by eliminating the need to collect and verify data gathered from various sources, which may differ substantially in their mode of generation and quality.

In this work, we have undertaken a substantial computational effort to generate a large, comprehensive polypeptide conformational energy data set using dispersion-corrected range-separated density-functional theory. The data set has several important features: 1) The conformational energies were obtained using a single computational method, which results in data with uniform quality; 2) The quality of the results obtained from the computational method we used to obtain the conformational energies is benchmarked against those obtained using complete-basis-set coupled-cluster methods. This provides a means for assessing the quality of our database; 3) The computational method we used to obtain conformational energies is of much higher quality than conventional force field methods used for large-scale protein modeling and is therefore fit for testing and parametrization of conventional force field methods. Therefore, our data can be used for molecular mechanics force field development^12–14^, and parametrization of cost-effective computational procedures like Atom-Centered Potentials (ACP)^15,16^ and other low-cost correction approaches^17–19^. It also serves as a direct source for comparative benchmark studies of various energy functions^20–27^, semi-empirical approaches^28–40^, and inexpensive electronic structure methods^41–47^ in the context of protein modeling.

## Methods

### Generation of the model geometries

**Figure 1:**
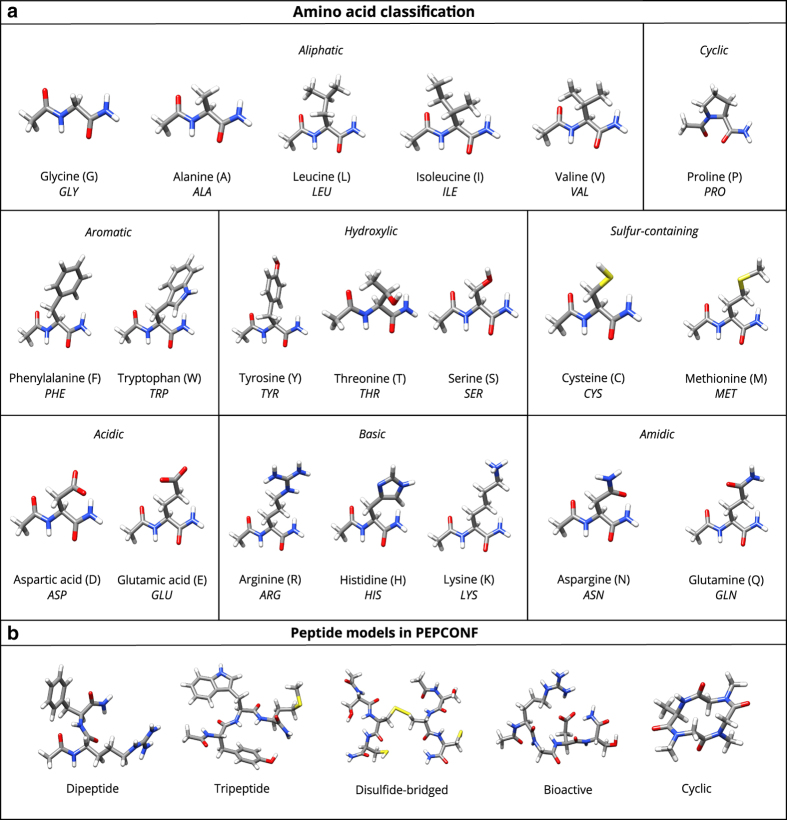
Molecular structure of the amino acids and representative peptide model systems considered in this work. (**a**) The classification of the twenty standard proteinogenic amino acids by the nature of their side-chains. The N-terminal and C-terminal are capped with acetyl and primary amide group, respectively. The single- and three-letter codes for each amino acid are also provided. (**b**) A representative candidate from each of the five different classes of peptide model systems considered in the PEPCONF data set.

The PEPCONF set comprises five different kinds of model systems:

- Dipeptides: All unique pairs of the twenty standard proteinogenic amino acids were selected (for instance, ALA-GLY and GLY-ALA were considered to be the same from the perspective of side chain-side chain interactions), leading to 136 neutral and 74 charged dipeptide geometries.
- Tripeptides: Unique combinations of tripeptide sequences were selected similarly but, in order to limit the number of combinations, one representative amino acid was chosen from each of the side-chain categories in Fig. 1a: Leucine for aliphatic, Proline for cyclic, Tryptophan for aromatic, Tyrosine for hydroxylic, Methionine for sulfur-containing, neutral Glutamic acid for acidic, Histidine for basic, and Glutamine for amidic side-chains. This yielded a total of 288 unique combinations of amino acid trimers.
- Disulfide-bridged: Oligopeptides where the two cysteine residues are internally connected via a disulfide bond (154 model systems).
- Bioactive: Oligopeptides where the chosen residue sequences were found to be associated with bio-functionality as reported in the literature^48^ (39 model systems).
- Cyclic: Oligopeptides where the N-terminus and C-terminus of the peptide backbone are connected to form a circular bond (64 model systems).

#### Structures

The initial gas-phase model geometries of the dipeptides, tripeptides, and bioactive peptides were generated using the *sequence* command in the *tleap* tool of *Amber16* software package^49–51^. The disulfide-bridged and cyclic peptides were generated manually from structures taken from the *Protein Data Bank* (PDB)^52,53^ and the *Cambridge Structural Database* (CSD)^54,55^, respectively. The N-terminal(s) and C-terminal(s) of all the representative model structures except for cyclic peptides were capped with acetyl (ACE) and primary amide (NHE) groups, respectively. The complete list of all the peptide structures considered in this work is provided in the supplementary file accompanying this article (Supplementary File 1).

The initial model geometries of disulfide-bridged oligopeptides were generated using an in-house fragmentation code and a combination of various *Amber16* tools like *pdb4amber*, *tleap*, and *pytleap*. Representative structures were initially obtained from searches of the *Protein Data Bank* (PDB) using the online advanced search interface with the following criteria: (i) only one disulfide bond, (ii) X-ray resolution between 2.5–3.5 Å, (iii) no modified polymeric residues, (iv) no free ligands, and (v) representative structures at 100% sequence identity. The resulting 191 hits were then processed with the *pdb4amber* tool to remove the water molecules from the PDB files and to select the most populous conformer. We then discarded 37 out of the 191 clean PDB files because the most populated conformer did not contain a disulfide bond. Finally, the clean PDB files were truncated using our fragmentation code and the disulfide-bridged cysteine residues of each model system were extracted along with at most four neighboring backbone residues. Each system was manually checked and then processed with *pytleap* and *tleap* to add the missing hydrogen atoms and terminal capping groups.

The initial model geometries of cyclic peptides were found using the *Conquest* software package to search for crystal structures in the *Cambridge Structural Database*. Cyclic sequences of proteinogenic amino acids were searched using the peptide building query tool. The following search criteria were used: (i) 3D coordinates must have been determined, (ii) R-factor less than or equal to 0.05, (iii) only non-disordered crystals, (iv) no errors present, (v) no ions present. The resulting structures were then exported to ‘mol2’ files which were converted to ‘xyz’ format using *Openbabel*^56,57^ and loaded in the *Avogadro*^58,59^ software package for visual inspection. Structures without a proper cyclic peptide backbone were not considered. Finally, the missing H-atoms were added using *Avogadro*.

The initial geometries of all the model systems, with the exception of cyclic peptides, were subjected to *Amber ff14SB*^21^ unconstrained force field energy relaxations using the *sander* module of *Amber16*.

#### Conformational search

A force field-based high-temperature molecular dynamics (HTMD) simulation approach^60^ was used in a manner similar to previous studies in the literature^61–64^ to generate the conformers for the non-cyclic peptides. Initial structures were subjected to canonical ensemble simulations with Langevin dynamics scaling at a temperature of 900 K. The MD steps were performed with the *sander* module of *Amber16* without solvent or periodicity. A heating (equilibration) step of 200 picoseconds was followed by a production run of 4.2 nanoseconds. Structures along the trajectory of the production run were sampled at uniform time intervals, resulting in 4000 conformers for each peptide model system. Each conformer was subjected to energy minimization using the *Amber ff14SB* force field.

The *Amber ff14SB* force field does not contain parameter for cyclic peptides. We therefore used the *RDKit* software package^65^ to generate cyclic peptide conformations. The accuracy and speed of *RDKit’s* conformer generation approach in comparison to other freely available conformer generation toolkits was reviewed in ref. 66, where it was reported that the program is suitable for less flexible molecules like the cyclic peptides considered in this work. A distance-geometry-based stochastic method^67^ was used to yield 100 conformers for each cyclic peptide. A very similar approach was recently used to generate the 3D conformations reported in the ANI-1 data set^68^.

#### Conformer binning strategy

The list of relaxed conformers was pruned using a binning strategy. Each set of non-cyclic conformers was sorted according to the force field energy, from most to least stable. The least stable conformers from the upper half of the list were removed, and the remainder of the list was divided into thirty equal energy intervals. From each interval, one conformer geometry was selected and was subjected to a single-point energy calculation with the BLYP gradient-corrected density functional^69,70^, and the 6-31 G^∗^ basis set^71,72^, combined with Grimme’s D3 dispersion-correction method^73,74^ with Becke-Johnson (BJ) damping function^75–81^ and recently developed basis set incompleteness potentials (BSIP)^82^. The calculations with the BLYP-D3(BJ)/6-31 G^∗^-BSIP level of theory were carried out using the *Gaussian* software package^83,84^, with SCF convergence criterion of 10^−6^ Hartrees and pruned integration grid with 99 radial and 590 angular points (ultrafine grid). The resulting BLYP-D3(BJ)/6-31 G^∗^-BSIP energies were used to select the six most stable conformers out of the thirty for entry into the PEPCONF data set.

In the case of the cyclic peptides, the 100 conformers generated by *RDKit* were geometry-optimized at the BLYP-D3(BJ)/MINIs-BSIP^69,70,73–82,85^ level of theory using the *Gaussian* package. The calculations employed SCF convergence criterion of 10^−8^ Hartrees, ultrafine integration grid, and the default optimization convergence criteria (maximum force = 4.5 × 10^−4^ Hartrees/Bohr, RMS force = 3 × 10^−4^ Hartrees/Bohr, maximum displacement = 1.8 × 10^−3^ Bohr, RMS displacement = 1.2 × 10^−3^ Bohr). The equilibrium geometries were sorted by energy and six conformations from equally-spaced energy intervals covering the whole energy range were then selected. The six conformations were then subjected to further geometry optimizations using BLYP-D3(BJ)/6-31 G^∗^-BSIP with the same SCF and grid settings as above and a ‘*verytight’* optimization convergence criteria (maximum force = 2 × 10^−6^ Hartrees/Bohr, RMS force = 1 × 10^−6^ Hartrees/Bohr, maximum displacement = 6 × 10^−6^ Bohr, RMS displacement = 4 × 10^−6^ Bohr).

### Generation of the reference energies

The PEPCONF data set contains 5 relative conformational energies (from the 6 conformations) for each peptide model system considered, yielding a total of 3775 data points and 4530 conformer structures. The reference energies were calculated with the LC-ωPBE^86,87^ range-separated density functional, and the aug-cc-pVTZ basis set of Dunning and co-workers^88–90^, combined with the exchange-hole dipole moment (XDM) dispersion-correction technique^75–81^. The rationale for this choice is that it offers a good compromise between accuracy and speed, and we expect range-separated hybrid functionals to minimize the impact of functional delocalization error on zwitterionic and charged species^91^. The resulting DFT-based approach was chosen as the reference level because of its excellent performance for gas-phase results of relative conformational energies (see Technical Validation).

A wave-function based approach like the “gold-standard” CCSD(T)/CBS would provide more reliable relative conformer energies^92,93^. However, CCSD(T)/CBS calculations are not feasible for the quite large systems (23–166 number of atoms) included in the data set. In addition, the PEPCONF data set is intended as a database for parametrization and benchmarking of force fields, semi-empirical methods and other low computational cost methods, which have much higher errors in conformational energies than those associated with LC-ωPBE-XDM/aug-cc-pVTZ. Future revisions of the PEPCONF set may become possible as computing power increases and approximate but accurate CCSD(T) methods are developed^94,95^.

### Code availability

The molecular dynamics simulations were carried out using *Amber16*, which is available from http://ambermd.org/ through a commercial license. The *Amber16* tools *pdb4amber*, *tleap*, and *pytleap* used for peptide structure editing and manipulation are part of the *Amber16* software package. The *Cambridge Structural Database 2018* and the *Conquest* program are distributed under a commercial license at https://www.ccdc.cam.ac.uk/. *RDKit* is an open-source cheminformatics software made available under the Berkeley Software Distribution (BSD) license at https://www.rdkit.org/. The *OpenBabel* software package was used for file-type interconversions and is freely available from http://openbabel.org/ under the GPL license. The *Avogadro* molecular editor and visualizer is an open-source program available at https://avogadro.cc/. The quantum-mechanical calculations were performed using the *Gaussian09/16* software packages, which can be purchased from Gaussian Inc. (http://gaussian.com/) under a commercial license. Finally, the Basis-Set Incompleteness Potentials (BSIP) for BLYP-D3(BJ)/MINIs and BLYP-D3(BJ)/6-31 G^∗^ level of theory can be obtained from the Supporting Information of ref. 82.

## Data Records

The conformational reference energies (in kcal/mol) and coordinates (in Å) of the conformer geometries present in the PEPCONF data set are publicly available free-of-charge from the Figshare (Data Citation 1) and GitHub (https://github.com/aoterodelaroza/pepconf) repositories in the plain-text DB-format described in Table 1. The atomic coordinates of the conformer geometries are also stored in a plain-text XYZ-format. The PEPCONF set contains five DB-format and six XYZ-format files for each peptide model system. In total, deposited files include 3775 DB-format files and 4530 XYZ-format files stored in their respective peptide classification directory named Dipeptide, Tripeptide, Disulfide, Bioactive, and Cyclic. A CSV-format file is also provided in each directory and contains the reference energy values for all the peptide systems in that directory.

### File format

For each molecule, the reference conformational energy, relative to the lowest-energy structure, and the atomic coordinates are stored in a file named *MoleculeName_A.db*, where A is the conformer identification number (1–5, ordered from lowest to highest relative energy). The Cartesian coordinates of the atoms are stored in files named *MoleculeName_B.xyz,* where B is 0–5 (ordered from lowest to highest relative energy), with 0 representing the lowest-energy reference structure.

The DB-format file contains a header line specifying the reference energy value (in kcal/mol) followed by two ‘*molc’* (short for molecule) blocks containing a unique integer identifier, charge, multiplicity, and the atomic coordinates (in Å) of the peptide conformer and its corresponding lowest energy conformer. The XYZ-format file contains a header line defining the number of atoms N, a comment line containing the charge and multiplicity, and N lines with each containing element type and X, Y, Z coordinates (in Å). The CSV-format file is a comma-separated plain-text file containing multiple lines and three columns. The columns are: (i) identification number, (ii) name of the peptide, and (iii) reference conformational energy (in kcal/mol).

## Technical Validation

The LC-ωPBE-XDM/aug-cc-pVTZ method was chosen as the reference level of theory for the single-point energy calculations of all the conformers in the PEPCONF data set. To justify the use of LC-ωPBE-XDM/aug-cc-pVTZ as the reference level, we checked its performance on several benchmark sets for conformational energies from the literature. The performance of LC-ωPBE-XDM/aug-cc-pVTZ is quantified in terms of the mean absolute error (MAE) relative to higher-level reference data. For Hobza’s 2008 conformer database of small peptides^1^, the MAE of LC-ωPBE-XDM/aug-cc-pVTZ relative to the CCSD(T)/CBS reference energies is 0.52 kcal/mol. The LC-ωPBE-XDM/aug-cc-pVTZ method also yields an MAE of 0.48 kcal/mol for the YMPJ^8^ set of amino acid conformers relative to the MP2-F12/cc-pVTZ-F12 + [CCSD(Ts)-F12b – MP2-F12]/cc-pVDZ-F12 data. The MAE of LC-ωPBE-XDM/aug-cc-pVTZ for the smaller peptide conformer sets are: 0.62 kcal/mol for CYCONF^4,11^ (relative to CCSD(T)/CBS), 0.61 kcal/mol for PCONF^2^ (relative to CCSD(T^∗∗^)-F12a/CBS) and 0.60 kcal/mol for TPCONF^3,5^ (relative to CCSD(T)/CBS).

Although they do not involve peptides, there are several other sets that can be used to validate the performance of LC-ωPBE-XDM/aug-cc-pVTZ for its ability to predict conformer energies. For example: 0.12 kcal/mol for ACONF^11,96^ (*n*-alkane conformations, relative to W1h-val), 0.07 kcal/mol for BUT14DIOL^97^ (conformations of butane-1,4-diol, relative to CCSD(T)-F12b/cc-pVTZ-F12), 0.75 kcal/mol for CCONF^98^ (conformations of glucose and α-maltose, relative to DLPNO-CCSD(T)/CBS), 0.21 kcal/mol for MCONF^99^ (melatonin conformations, relative to CCSD(T)/CBS), 0.24 kcal/mol for SCONF^11,100^ (sugar conformations, relative to CCSD(T)/CBS), and 0.62 kcal/mol for UpU46^101^ (RNA backbone conformations, relative to DLPNO-CCSD(T)/CBS). For comparison with peptide based non-covalent interaction energy data sets, LC-ωPBE-XDM/aug-cc-pVTZ gives MAE of 0.33 and 0.23 kcal/mol relative to DW-CCSD(T)-F12/aug-cc-pV(D + d)z for the BBI^102^ and SSI^102^ sets of backbone-backbone and sidechain-sidechain interactions, respectively. LC-ωPBE-XDM/aug-cc-pVTZ also yields an MAE of 0.28 and 0.18 kcal/mol for the S22 and S66 sets and 0.23 and 0.15 kcal/mol for the S22x5 and S66x8 sets of non-covalent binding energies calculated at the CCSD(T)/CBS limit, respectively^103–107^. A detailed analysis of the LC-ωPBE-XDM/aug-cc-pVTZ method for non-covalent interactions and thermochemistry can also be found in ref. 108.

## Additional information

**How to cite this article**: Prasad, V. K. *et al.* PEPCONF, a diverse data set of peptide conformational energies. *Sci. Data*. 6:180310 doi: 10.1038/sdata.2018.310 (2019).

**Publisher’s note**: Springer Nature remains neutral with regard to jurisdictional claims in published maps and institutional affiliations.

## Supplementary Material



Supplementary File 1

## Figures and Tables

**Table 1:** A description of the DB-format file or the database-file format (.db) for a peptide system containing N number of atoms.

Line	Column	Content
1	1	‘ref’ string specifying the reference energy
1	2	reference conformational energy (peptide A - peptide B) (in kcal/mol)
2	1	‘molc’ string specifying start of the first molecular block
2	2	unique integer identifier, 1 indicating peptide conformer A
2	3	charge of the conformer A
2	4	multiplicity of the conformer A
3, …, N + 2	1	element type
3, …, N + 2	2	X coordinates (in Å)
3, …, N + 2	3	Y coordinates (in Å)
3, …, N + 2	4	Z coordinates (in Å)
N + 3	1	‘end’ string specifying end of the first molecular block
N + 4	1	‘molc’ string specifying start of the second molecular block
N + 4	2	unique integer identifier, −1 indicating peptide conformer B (with lower energy than A)
N + 4	3	charge of the peptide conformer B
N + 4	4	multiplicity of the peptide conformer B
N + 4, …, 2 N + 4	1	element type
N + 4, …, 2 N + 4	2	X coordinates (in Å)
N + 4, …, 2 N + 4	3	Y coordinates (in Å)
N + 4, …, 2 N + 4	4	Z coordinates (in Å)
2 N + 5	1	‘end’ string specifying end of the second molecular block
