# Supplementary material for: PEPCONF, a diverse data set of peptide conformational energies
Source: Sci Data. 2019 Jan 22;6:180310. doi: 10.1038/sdata.2018.310 (PMC6343515; doi:10.1038/sdata.2018.310)
Supplement: Supplementary File 1 [file sdata2018310-s2.docx]

**Supplementary File 1**

**PEPCONF, a diverse data set of peptide conformational energies**

Viki Kumar Prasad^a^, Alberto Otero-de-la-Roza^b,*^, Gino A. DiLabio^a,c,*^

1. Department of Chemistry, University of British Columbia, Okanagan, 3247 University Way, Kelowna, British Columbia, Canada V1V 1V7
2. Department of Physical and Analytical Chemistry, Faculty of Chemistry, University of Oviedo, Oviedo, Spain 33006
3. Faculty of Management, University of British Columbia, Okanagan, 1137 Alumni Avenue, Kelowna, British Columbia, Canada V1V 1V7

The 210 dipeptide sequences considered for this work in terms of their three letter amino acid codes are as follows:

ALA-ALA, ALA-ASN, ALA-CYS, ALA-GLN, ALA-GLY, ALA-HIS, ALA-ILE, ALA-LEU, ALA-MET, ALA-PHE,

ALA-PRO, ALA-SER, ALA-THR, ALA-TRP, ALA-TYR, ALA-VAL, ARG-ALA, ARG-ARG, ARG-ASN, ARG-CYS, ARG-GLN, ARG-GLY, ARG-HIS, ARG-ILE, ARG-LEU, ARG-LYS, ARG-MET, ARG-PHE, ARG-PRO, ARG-SER, ARG-THR, ARG-TRP, ARG-TYR, ARG-VAL, ASN-ASN, ASN-CYS, ASN-GLN, ASN-MET, ASP-ALA, ASP-ARG, ASP-ASN, ASP-ASP, ASP-CYS, ASP-GLN, ASP-GLU, ASP-GLY, ASP-HIS, ASP-ILE, ASP-LEU, ASP-LYS, ASP-MET, ASP-PHE, ASP-PRO, ASP-SER, ASP-THR, ASP-TRP, ASP-TYR, ASP-VAL, CYS-CYS, CYS-MET, GLN-CYS, GLN-GLN, GLN-MET, GLU-ALA, GLU-ARG, GLU-ASN, GLU-CYS, GLU-GLN, GLU-GLU, GLU-GLY, GLU-HIS, GLU-ILE, GLU-LEU, GLU-LYS, GLU-MET, GLU-PHE, GLU-PRO, GLU-SER, GLU-THR, GLU-TRP, GLU-TYR, GLU-VAL, GLY-ASN, GLY-CYS, GLY-GLN, GLY-GLY, GLY-HIS, GLY-ILE, GLY-LEU, GLY-MET, GLY-PHE, GLY-PRO, GLY-SER, GLY-THR, GLY-TRP, GLY-TYR, GLY-VAL, HIS-ASN, HIS-CYS, HIS-GLN, HIS-HIS, HIS-MET, HIS-SER, HIS-THR, ILE-ASN, ILE-CYS, ILE-GLN, ILE-HIS, ILE-ILE, ILE-LEU, ILE-MET, ILE-PHE, ILE-PRO, ILE-SER, ILE-THR, ILE-TRP, ILE-TYR, ILE-VAL, LEU-ASN, LEU-CYS, LEU-GLN, LEU-HIS, LEU-LEU, LEU-MET, LEU-PHE, LEU-PRO, LEU-SER, LEU-THR, LEU-TRP, LEU-TYR, LEU-VAL, LYS-ALA, LYS-ASN, LYS-CYS, LYS-GLN, LYS-GLY, LYS-HIS, LYS-ILE, LYS-LEU, LYS-LYS, LYS-MET, LYS-PHE, LYS-PRO, LYS-SER, LYS-THR, LYS-TRP, LYS-TYR, LYS-VAL, MET-MET, PHE-ASN, PHE-CYS, PHE-GLN, PHE-HIS, PHE-MET, PHE-PHE, PHE-SER, PHE-THR, PHE-TRP, PHE-TYR, PRO-ASN, PRO-CYS, PRO-GLN, PRO-HIS, PRO-MET, PRO-PHE, PRO-PRO, PRO-SER, PRO-THR, PRO-TRP, PRO-TYR, PRO-VAL, SER-ASN, SER-CYS, SER-GLN, SER-MET, SER-SER, SER-THR, THR-ASN, THR-CYS, THR-GLN, THR-MET, THR-THR, TRP-ASN, TRP-CYS, TRP-GLN, TRP-HIS, TRP-MET, TRP-SER, TRP-THR, TRP-TRP, TRP-TYR, TYR-ASN, TYR-CYS, TYR-GLN, TYR-HIS, TYR-MET, TYR-SER, TYR-THR, TYR-TYR, VAL-ASN, VAL-CYS, VAL-GLN, VAL-HIS, VAL-MET, VAL-PHE, VAL-SER, VAL-THR, VAL-TRP, VAL-TYR, VAL-VAL.

The 288 tripeptide sequences considered for this work in terms of their three letter amino acid codes are as follows:

GLH-GLH-GLH, GLH-GLN-GLH, GLH-HIS-GLH, GLH-LEU-GLH, GLH-MET-GLH, GLH-PRO-GLH, GLH-TRP-GLH, GLH-TYR-GLH, GLN-GLH-GLH, GLN-GLH-GLN, GLN-GLH-HIS, GLN-GLH-MET, GLN-GLN-GLH, GLN-GLN-GLN, GLN-GLN-HIS, GLN-GLN-MET, GLN-HIS-GLH, GLN-HIS-GLN, GLN-HIS-HIS, GLN-HIS-MET, GLN-LEU-GLH, GLN-LEU-GLN, GLN-LEU-HIS, GLN-LEU-MET, GLN-MET-GLH, GLN-MET-GLN, GLN-MET-HIS, GLN-MET-MET, GLN-PRO-GLH, GLN-PRO-GLN, GLN-PRO-HIS, GLN-PRO-MET, GLN-TRP-GLH, GLN-TRP-GLN, GLN-TRP-HIS, GLN-TRP-MET, GLN-TYR-GLH, GLN-TYR-GLN, GLN-TYR-HIS, GLN-TYR-MET, HIS-GLH-GLH, HIS-GLH-HIS, HIS-GLN-GLH, HIS-GLN-HIS, HIS-HIS-GLH, HIS-HIS-HIS, HIS-LEU-GLH, HIS-LEU-HIS, HIS-MET-GLH, HIS-MET-HIS, HIS-PRO-GLH, HIS-PRO-HIS, HIS-TRP-GLH, HIS-TRP-HIS, HIS-TYR-GLH, HIS-TYR-HIS, LEU-GLH-GLH, LEU-GLH-GLN, LEU-GLH-HIS, LEU-GLH-LEU, LEU-GLH-MET, LEU-GLH-PRO, LEU-GLH-TRP, LEU-GLH-TYR, LEU-GLN-GLH, LEU-GLN-GLN, LEU-GLN-HIS, LEU-GLN-LEU, LEU-GLN-MET, LEU-GLN-PRO, LEU-GLN-TRP, LEU-GLN-TYR, LEU-HIS-GLH, LEU-HIS-GLN, LEU-HIS-HIS, LEU-HIS-LEU, LEU-HIS-MET, LEU-HIS-PRO, LEU-HIS-TRP, LEU-HIS-TYR, LEU-LEU-GLH, LEU-LEU-GLN, LEU-LEU-HIS, LEU-LEU-LEU, LEU-LEU-MET, LEU-LEU-PRO, LEU-LEU-TRP, LEU-LEU-TYR, LEU-MET-GLH, LEU-MET-GLN, LEU-MET-HIS, LEU-MET-LEU, LEU-MET-MET, LEU-MET-PRO, LEU-MET-TRP, LEU-MET-TYR, LEU-PRO-GLH, LEU-PRO-GLN, LEU-PRO-HIS, LEU-PRO-LEU, LEU-PRO-MET, LEU-PRO-PRO, LEU-PRO-TRP, LEU-PRO-TYR, LEU-TRP-GLH, LEU-TRP-GLN, LEU-TRP-HIS, LEU-TRP-LEU, LEU-TRP-MET, LEU-TRP-PRO, LEU-TRP-TRP, LEU-TRP-TYR, LEU-TYR-GLH, LEU-TYR-GLN, LEU-TYR-HIS, LEU-TYR-LEU, LEU-TYR-MET, LEU-TYR-PRO, LEU-TYR-TRP, LEU-TYR-TYR, MET-GLH-GLH, MET-GLH-HIS, MET-GLH-MET, MET-GLN-GLH, MET-GLN-HIS, MET-GLN-MET, MET-HIS-GLH, MET-HIS-HIS, MET-HIS-MET, MET-LEU-GLH, MET-LEU-HIS, MET-LEU-MET, MET-MET-GLH, MET-MET-HIS, MET-MET-MET, MET-PRO-GLH, MET-PRO-HIS, MET-PRO-MET, MET-TRP-GLH, MET-TRP-HIS, MET-TRP-MET, MET-TYR-GLH, MET-TYR-HIS, MET-TYR-MET, PRO-GLH-GLH, PRO-GLH-GLN, PRO-GLH-HIS, PRO-GLH-MET, PRO-GLH-PRO, PRO-GLH-TRP, PRO-GLH-TYR, PRO-GLN-GLH, PRO-GLN-GLN, PRO-GLN-HIS, PRO-GLN-MET, PRO-GLN-PRO, PRO-GLN-TRP, PRO-GLN-TYR, PRO-HIS-GLH, PRO-HIS-GLN, PRO-HIS-HIS, PRO-HIS-MET, PRO-HIS-PRO, PRO-HIS-TRP, PRO-HIS-TYR, PRO-LEU-GLH, PRO-LEU-GLN, PRO-LEU-HIS, PRO-LEU-MET, PRO-LEU-PRO, PRO-LEU-TRP, PRO-LEU-TYR, PRO-MET-GLH, PRO-MET-GLN, PRO-MET-HIS, PRO-MET-MET, PRO-MET-PRO, PRO-MET-TRP, PRO-MET-TYR, PRO-PRO-GLH, PRO-PRO-GLN, PRO-PRO-HIS, PRO-PRO-MET, PRO-PRO-PRO, PRO-PRO-TRP, PRO-PRO-TYR, PRO-TRP-GLH, PRO-TRP-GLN, PRO-TRP-HIS, PRO-TRP-MET, PRO-TRP-PRO, PRO-TRP-TRP, PRO-TRP-TYR, PRO-TYR-GLH, PRO-TYR-GLN, PRO-TYR-HIS, PRO-TYR-MET, PRO-TYR-PRO, PRO-TYR-TRP, PRO-TYR-TYR, TRP-GLH-GLH, TRP-GLH-GLN, TRP-GLH-HIS, TRP-GLH-MET, TRP-GLH-TRP, TRP-GLH-TYR, TRP-GLN-GLH, TRP-GLN-GLN, TRP-GLN-HIS, TRP-GLN-MET, TRP-GLN-TRP, TRP-GLN-TYR, TRP-HIS-GLH, TRP-HIS-GLN, TRP-HIS-HIS, TRP-HIS-MET, TRP-HIS-TRP, TRP-HIS-TYR, TRP-LEU-GLH, TRP-LEU-GLN, TRP-LEU-HIS, TRP-LEU-MET, TRP-LEU-TRP, TRP-LEU-TYR, TRP-MET-GLH, TRP-MET-GLN, TRP-MET-HIS, TRP-MET-MET, TRP-MET-TRP, TRP-MET-TYR, TRP-PRO-GLH, TRP-PRO-GLN, TRP-PRO-HIS, TRP-PRO-MET, TRP-PRO-TRP, TRP-PRO-TYR, TRP-TRP-GLH, TRP-TRP-GLN, TRP-TRP-HIS, TRP-TRP-MET, TRP-TRP-TRP, TRP-TRP-TYR, TRP-TYR-GLH, TRP-TYR-GLN, TRP-TYR-HIS, TRP-TYR-MET, TRP-TYR-TRP, TRP-TYR-TYR, TYR-GLH-GLH, TYR-GLH-GLN, TYR-GLH-HIS, TYR-GLH-MET, TYR-GLH-TYR, TYR-GLN-GLH, TYR-GLN-GLN, TYR-GLN-HIS, TYR-GLN-MET, TYR-GLN-TYR, TYR-HIS-GLH, TYR-HIS-GLN, TYR-HIS-HIS, TYR-HIS-MET, TYR-HIS-TYR, TYR-LEU-GLH, TYR-LEU-GLN, TYR-LEU-HIS, TYR-LEU-MET, TYR-LEU-TYR, TYR-MET-GLH, TYR-MET-GLN, TYR-MET-HIS, TYR-MET-MET, TYR-MET-TYR, TYR-PRO-GLH, TYR-PRO-GLN, TYR-PRO-HIS, TYR-PRO-MET, TYR-PRO-TYR, TYR-TRP-GLH, TYR-TRP-GLN, TYR-TRP-HIS, TYR-TRP-MET, TYR-TRP-TYR, TYR-TYR-GLH, TYR-TYR-GLN, TYR-TYR-HIS, TYR-TYR-MET, TYR-TYR-TYR.

The 154 four-character Protein Data Bank (PDB) codes from which the disulfide-bridged oligopeptides were extracted are as follows:

1a43, 1aum, 1avp, 1baj, 1bmg, 1bvo, 1bwz, 1c8e, 1eha, 1eia, 1emr, 1f02, 1f6l, 1gku, 1ijs, 1jjh, 1k5h, 1kac, 1ml8, 1mqa, 1mry, 1nov, 1ny7, 1nyl, 1ou5, 1p5y, 1pfc, 1plr, 1q7q, 1qb3, 1qe0, 1qfp, 1rlr, 1ry7, 1s0g, 1s94, 1se2, 1t3b, 1tgo, 1tjd, 1tmf, 1vb2, 1ver, 1vkx, 1wcs, 1xyh, 1zmw, 1zzd, 2a1r, 2a1s, 2a8z, 2ayu, 2cas, 2czk, 2duk, 2ecf, 2h2y, 2h4m, 2h4r, 2if9, 2irm, 2lve, 2mha, 2o8v, 2ot8, 2ov8, 2p62, 2q2p, 2q98, 2r30, 2vaj, 2w2s, 2wxw, 2wzr, 2xpe, 2yyn, 2z1b, 2z5j, 2z8h, 2z9s, 2zf8, 3a0f, 3b3l, 3b43, 3ceq, 3ebm, 3fte, 3hpm, 3hxq, 3i7k, 3ikk, 3pin, 3psi, 3q2c, 3rg6, 3tn9, 3uj1, 3uw0, 3v6y, 3zor, 4az8, 4c85, 4d2g, 4dbg, 4dks, 4eig, 4fi9, 4ga7, 4i6j, 4jgy, 4jup, 4jvy, 4k0r, 4kce, 4lgs, 4lpz, 4nc2, 4nik, 4q4j, 4q5y, 4qrr, 4tkn, 4tlw, 4uy9, 4wnf, 4xfu, 4yzy, 4zyh, 5c4r, 5cca, 5cfc, 5cyu, 5d0o, 5d06, 5eta, 5eve, 5feg, 5h07, 5hpc, 5i50, 5id4, 5j6e, 5jhf, 5k23, 5k93, 5kud, 5l7c, 5lad, 5lsk, 5mqo, 5omn, 5sv7, 5tjw, 5wco.

The 64 Cambridge Structural Database (CSD) codes of the cyclic peptides that were considered in this work are as follows:

AAGAGG10, AAGGAG10, ALASAR, ALPRAL10, BIHTUH, BIHXUL10, BINJIR, BUYXOI, CACNOJ10, CAHWEN, CAMVES, CEWCIQ10, CGDLLL10, CGLEGL, CGLPGL, CGPGAP10, CLPGDH, CYBGPP, CYHEXG, DASXIE, DEWFEQ, DICWET, DUPKEE, DUTLAF10, DUVGOQ10, DUYTIA, EVAPUM, FIVSAE, GAJFAY, GEHKUC, GGAAGG, GICHOP, GIPKAR10, GOKXOV, JUXHAL, KARPIE, KIVDIC, LENKIY, LETHIE, LETPIM, LEYCAV, NIWHEH, NUCZUH, NUWNEY, PAPGAP, PAPRVA, POWWEE, PROGLY20, RUQVAB, SAFVOM, SEFTIG, SOWGOA, TALVAD, UBADEJ, UNONES, UZUKUW, VAWTAQ, WUYGII, YEXJIV, YOMNOE, ZAJPAB, ZEHDEV, ZOHMIS, ZUKRAY.

The 39 bioactive peptide sequences in terms of their one letter amino acid codes as well as the associated bio-functionality as reported in literature are as follows:

| *Peptide sequence* | *Bio-functionality^[[1]](#footnote-1)^* |
| --- | --- |
| RGD, RGDS, KGD, PHSRN, REDV, YIGSR, IKVAV, PDSGR, DGEA, KRSR, GFPGER | Cell adhesion peptides |
| (GAGA)_2_, (GPP)_2_ | Structural peptides |
| CNGRC, CGKRK, CRGDK, CREKA | Anti-tumor peptides |
| RRWWRF, FRWWHR, KLAK | Anti-microbial peptides |
| VYIHPF, WMNF | Peptide hormone |
| GPQGIAG, APGL, VRN | Tissue-engineering application |
| GNNQQNY, VQIVYK, NFGAIL, KLVFF, KLVFFAE, LPFFD, FEFEFKEK | Model amyloid peptides |
| YGGFM, YGGFL, YPWF, YPFF | Neuropeptides |
| RLNVY, RLGVY | Immune-related peptides |
| HHHHHH | Protein tags |

1. Hamley, I. W. Small bioactive peptides for biomaterials design and therapeutics. *Chem. Rev.* **117,** 14015–14041 (2017). [↑](#footnote-ref-1)
